# The Temporality of Aberrant Salience and Schizophrenia

**DOI:** 10.3389/fnint.2022.925716

**Published:** 2022-07-11

**Authors:** David H. V. Vogel

**Affiliations:** Department of Psychiatry and Psychotherapy, Faculty of Medicine and University Hospital Cologne, University of Cologne, Cologne, Germany

**Keywords:** temporality, time experience, aberrant salience, schizophrenia, predictive processing, psychopathology

## Introduction

As with other medical conditions, mental disorders and illness are defined by the symptoms exhibited by the patient and inquirable by the health care professional (World Health Organization, 1992; American Psychiatric Association, 2013; Casey and Kelly, 2019). Diagnostic procedures aim to work out these symptoms in patients and healthcare system users and arrange them into individual syndromes.

In the case of mental disorders, the assessment of most signs and symptoms currently falls within the area of competence of descriptive psychopathology (Berrios, 1984; Oyebode, 2008; Taylor and Vaidya, 2008; Casey and Kelly, 2019). Most clinicians know psychopathology as a method primarily directed at symptom description. Importantly, in clinical practice, symptom description necessarily takes place in a semi-structured interview between the practitioner and their patient. In the ensuing conversation, the clinician aims at understanding patients' complaints and symptoms as an expression of the underlying disorder (Stanghellini, 2009, 2010).

The arguably predominant natural science of psychiatry is (clinical) neuroscience. As with other medical disciplines, the theories tested by neuroscientists aim at explaining mental disorders as resulting from corresponding disorders of the brain. While many competing neuroscientific theories exist which attempt at explaining the different mental conditions treated by psychiatrists and psychotherapists, the proposal that differences in the predictive processes of the brain cause the emergence of psychopathological symptoms has gained considerable traction over recent years (Fletcher and Frith, 2009; Hohwy et al., 2015; Vogel et al., 2019a,b; Fabry, 2020; Kube et al., 2020).

In his pioneering work “General Psychopathology” German psychopathologist Karl Jaspers pointed out the dichotomy between psychopathology and (neuro-) science as the two complementary sides of research in psychiatry (Jaspers, 1913, 1997). More important than this obvious distinction is the distinction between both disciplines' methods (Jaspers, 1913). While psychopathology primarily—but not exclusively—relies on “understanding” (Verstehen) a patient experience, neuroscientific methods aim at “explaining” (Erklären) it through causal and replicable relationships with and between brain states (Jaspers, 1913, pp. 13 ff., pp.145 ff., and pp. 189 ff.). The focus on a methodological delimitation emphasizes the complementary nature of psychiatric research approaches (Jaspers, 1913, pp. 4 ff.). Ideally, the knowledge gained through these different methods converges and informs the other.

Against this background, the case of schizophrenia poses a particularly interesting example of insights from neuroscience enriching and substantially extending theories from phenomenological psychopathology. While several theories on the biological causes of the disorder exist, one of the most prominent is that of aberrant salience (Kapur, 2003; Kapur et al., 2005). Briefly, it states that the dopaminergic neural mechanisms underlying the perception of motivating stimuli changes during acute states of schizophrenia. Signs and symptoms of acute psychosis in schizophrenia can then be explained as secondary results from this aberrant salience. Informed by research on perception and experience as predictions generated by the brain (Friston, 2009, 2010; Hohwy, 2013, 2018), the theory of prediction errors as causative to many primary symptoms of schizophrenia has emerged from the aberrant salience theory (Fletcher and Frith, 2009; Lalanne et al., 2010; Adams et al., 2015). Recent work in psychopathology has raised the theory of an altered temporal experience in schizophrenia (Minkowski, 1933; Vogeley and Kupke, 2006; Fuchs, 2007, 2013; Stanghellini et al., 2015; Moskalewicz, 2016; Fuchs and Van Duppen, 2017; Vogel et al., 2019a). Both the consideration from psychopathology and that from neuroscience converge on one common core concept: a disturbance in temporality.

In the following, we will first give account of the mentioned theories, namely aberrant salience and altered time experience in schizophrenia. Subsequently, we will elaborate temporality as the common ground of both concepts. In conclusion, we will demonstrate how both positions inform and complement one another where the other reaches its methodological limits.

## Aberrant Salience, Predictive Processing, and Psychosis

### Salience Expectations

In a brain unaffected by schizophrenic psychosis, midbrain dopamine activity encodes for the prediction of reward (Kapur, 2003; Kapur et al., 2005). An increase in neural dopamine release correlates with the anticipation of an event (Fiorillo et al., 2003; Dreher et al., 2009; Salimpoor et al., 2011). This reward prediction works across various time scales. Dopamine release may encode for expected immediate action consequences, hence facilitating learning (Wise, 2004). Dopamine release may also encode for anticipated events minutes away (Berridge and Robinson, 1998; Kapur et al., 2005; Shohamy and Adcock, 2010), correlating with motivation and enabling goal directed behavior.

The motivational aspect of dopamine release corresponds to motivational salience (Berridge and Robinson, 1998; Kapur, 2003; Kapur et al., 2005). The term describes the neural system's ability to detect novel external stimuli, to reach them by appropriate behavior, and to accomplish successful (inter-) action. Importantly, salience is not the result of goal directed actions, but the prerequisite to their onset. In this sense, salience is a prediction of future potential reward and gratification. Dopamine activity assumedly encodes for these reward predictions.

Reward prediction connects dopamine and salience to predictive processes in general. More recent theory assumes that the neural apparatus works in the sense of Bayesian predictive processing, having coined the term “Bayesian Brain” (Friston, 2009, 2010). The brain's predictive processes function by calculating expected outcome from perceptual input. The calculations are however, not performed in isolation, but influenced by information acquired by the neural system earlier. The perceptual information functions as the bottom-up input, while the system's prior knowledge is called the top-down information (Friston, 2010). The aim of this system is to minimize the potential error in the prediction of oncoming events (Fletcher and Frith, 2009; Friston, 2010). The system continuously compares the current input with its prior prediction. Whenever prediction errors occur, the system automatically updates itself to improve future predictions (Fletcher and Frith, 2009; Friston, 2009, 2010).

Although many occurrences in the natural environment may thus be accurately predicted, variance is a natural occurrence in our environment. Small and negligible errors in prediction do not necessarily require a change in future prediction. Accordingly, the Bayesian Brain needs to encode in its predictive process the probability of change. The system encodes for its own precision. This encoding is believed to be a function of dopamine activity (Corlett et al., 2010; Friston et al., 2012).

In summary, the predictive process contains three parts. The prior prediction, the prediction error, and the posterior update of prediction. The relationship between the three aspects is mediated by dopaminergic activity as corresponding to the probability of the predictive process's accuracy.

Determined by precision and prediction error, the system compares the perceptual bottom-up information to a top-down prediction. This may result in a new prediction error. The system again passes the new error up its hierarchy, comparing it to the next top-down prediction. It repeats this process, until the error is accounted for (Friston, 2009). Put simply, whenever the system perceives something that does not fit with its predicted expectation, it attempts at explaining why the prediction was inaccurate.

This demonstrates the significance of dopamine and salience in the predictive process. Hypothetically speaking, the dopaminergic firing of neurons encodes for the change the system should expect and be ready for (Friston et al., 2012; Hohwy et al., 2015). It relates to the assumed inaccuracy of the prediction. To account for the environment's variance and variability the organism automatically assumes and (dopaminergically) encodes that it may be wrong about its predictions. The predetermined expectation of inaccuracy in the environment reflects the system's built for change in the environment.

Salience expectations also reflect assumed change. In the sense of predictive processing, the system increases the assumption of change due to a perceptual input of new or unexpected event or object. The corresponding top-down predictions mark the salient percept as a meaningful goal to facilitate adequate (inter-) action.

### Aberrant Salience and Impaired Prediction Error Evaluation

The aberrant salience hypothesis (Kapur et al., 2005) attempts at explaining schizophrenia and its symptoms as caused by a dysregulated release of dopamine in the brain. From its regulated, phasic state, the dopaminergic system assumably becomes highly responsive and dysphasic (Grace, 2016; Sonnenschein et al., 2020). The resulting release causes random events to appear novel and meaningful (Kapur et al., 2005).

In terms of a change in predictive processing (Fletcher and Frith, 2009), the system constantly assumes that its predictions are faulty. Prediction errors are consequently judged to be of great relevance and hence salience. Errors then follow the hierarchy, which tries to explain them by updating top-down predictors. The false evaluation and updating on the different levels of the hierarchy then causes the varying symptoms of schizophrenia, ranging from perceptual disturbances to delusion (Fletcher and Frith, 2009; Adams et al., 2015; Sterzer et al., 2018).

A person in an acute state of schizophrenia might hence perceive unusual unforeseeable events and objects of sudden significance. Hallucinations and delusions may then be understood as the consequence of the neural system “explaining-away” of this sudden significance (Friston, 2010). If a person believes to be persecuted by seemingly random bystanders, aberrant salience theory would suggest that the cause of this experience is the random firing of dopaminergic neurons providing these bystanders and the otherwise innocent situation with unexpected meaning. Own thoughts and experiences thus enriched with a quality of novelty may lead to hallucinatory experiences and sense of self-disorders.

## Temporal Synthesis and Altered Time Experience

### The Temporality of Experience

The psychopathology of time experience and temporality is founded in the phenomenology of time. The theories latter adopted by psychopathologists, concern two aspects of temporality: Biographical time, also referred to as narrative time (Kupke, 2009; Fuchs, 2013) or the macro layer of time (Vogel et al., 2020a); and intentional time (Kupke, 2009; Fuchs, 2013), or micro-layer of time (Vogel et al., 2020a). While the former assumably plays a larger role in mental disorders such as major depression (Straus, 1928; Fuchs, 2001; Kupke, 2009; Stanghellini et al., 2017; Vogel et al., 2018) it is intentional time which is usually discussed as altered in schizophrenia.

In this section, we will attempt to briefly outline both aspects of time. As most theory on time and schizophrenia focuses on intentional time, we will put some focus on this concept. We will explain how it can be transferred into a theory on schizophrenia in the following Section Fragmented Time Experience.

Biographical time, also referred to as narrative time (Kupke, 2009; Fuchs, 2013) or the macro layer of time (Vogel et al., 2020a) is the conscious layer of our experience where we remember our past, live our present, and plan our future. It is also, where we experience events approaching and time as passing. We structure our biographical time by means of telling our life story (Ricoeur, 1980) and assign meaning to specific events based on that story.

The second layer of time is intentional time (Kupke, 2009; Fuchs, 2013), or micro-layer of time (Vogel et al., 2020a). Where narrative time forms the biographical present, intentional time forms the “now”. Simultaneously, it composes the prereflective, automatic synthesis of time as a temporal flow, as a projection toward the future. Husserl famously conceptualized this “passive synthesis” of now as emerging from what he referred to as retention, (primal) impression, and protention (Husserl, 1928, 2019). It is distinct from the “active synthesis” of the narrative, actively performed by the individual. Retention, impression, and protention automatically (passively) make the present moment and guarantee the possibility of experiencing it as a temporal unity. It is hence the preconscious prerequisite to any active synthesis.

In simple terms, passive time synthesis describes the way in which human consciousness appears as coherent and moving forward. The theory poses that at any given moment, we experience time as a continuum. A current perception can only make sense as long as its experience entails information about the percept just past and the perceptual input about to follow. Husserl's famous example is listening to a melody (Husserl, 1928). What we perceive while listening to a piece of music are the notes being played. However, we experience not just the notes, but the notes as part of a melody. Such an experience is only possible if the current percept contains a reference about the last note(s) we heard, and information about the note(s) about to resound.

This means that—just like narrative time (past, present, future)—the present moment has a tripartite structure: retention, impression, and protention. The knowledge or information about the percept(s) *just past* is referred to as retention. The percept in consciousness *right now* is the impression. Relating to the percept(s) *just about* to be played it is called protention (Husserl, 1928).

Retention refers to the past, present in the current experiential moment. It is not to be confused with memory. In the current impression, the past is present as the retained former impressions. Accordingly, consciousness inherently entails the past and an experience can never be fully lost. With each novel impression, the retention becomes more and more past, until it drops past the “horizon” of consciousness (Husserl, 1928; Dainton, 2010).

Protention refers to the future anticipated by consciousness. It is specifically open toward the future making possible the anticipation of the future (Husserl, 1928). Just as with retention, the future lies behind a “horizon”. With each new emerging primal impression, events draw nearer in protention and become more and more likely to occur. As with retention, the acceptance of the necessary occurrence of a future is inherent to consciousness (Husserl, 1928; Dainton, 2010).

According to the Husserlian concept, only if the past and the future are present in the right-now, is it possible to experience events with or changes over a duration. Passive synthesis generates a “temporal field” (Zeithof) (Husserl, 1928, p 33; Kupke, 2009, pp. 55 ff.) which constitutes the present of the experiential moment. Thus temporally extended, the present continuously changes and moves forward in time. In this continuous flow of impressions lies the future-directedness and intentionality of all experiencing (Husserl, 1928; Dainton, 2010; Vogel et al., 2020a). For Husserl this entails both the prerequisite subject intentionality—the continuity of the conscious experience [longitudinal intentionality (Längsintentionalität)]—as well as object intentionality—the continuity of the experienced object [transverse intentionality (Querintentionalität)] (Husserl, 1928; Dainton, 2010). Both aspects of this “double intentionality” (Husserl, 1928; Dainton, 2010) are inseparably linked.

### Fragmented Time Experience

In schizophrenia, time experience is “fragmented”, or “disarticulated” (Vogeley and Kupke, 2006; Fuchs, 2007, 2013; Stanghellini et al., 2015; Fuchs and Van Duppen, 2017; Vogel et al., 2019a). The initial texts on the subject consider a “weakening in the protentional function” as the phenomenon underlying this fragmentation of experience (Fuchs, 2007; Fuchs and Van Duppen, 2017). Protention insufficiently anticipates the ongoing temporal events. Consciousness does not reliably judge experiences' probabilities. The temporal synthesis fails and “gaps” open within it. Events within these gaps intrude into the temporal field as unforeseen or unusual. The patients' “basic self-coherence” is altered (Vogeley and Kupke, 2006).

In short, the theory of “fragmented/disarticulated time” (Vogeley and Kupke, 2006; Fuchs, 2007, 2013; Stanghellini et al., 2015; Fuchs and Van Duppen, 2017; Vogel et al., 2019a) proposes that the forward directed process of protention does no longer provide the most probable experience as about to occur. In its place falls the assumed gap. Obviously, perception still occurs. An unanticipated event occurs within the gap. As it has not previously been part of protention, this event lacks the substance of having been part of the prior experience. It must seem to come out of nowhere. Accordingly, the unprotended event carries the experience of imminence and novelty (Fuchs, 2007; Fuchs and Van Duppen, 2017; Vogel et al., 2019a).

An experience manifesting itself in consciousness in such a way comes as a surprise for the experiencing person (Fuchs, 2007; Fuchs and Van Duppen, 2017; Vogel et al., 2019a). In other words, it lacks the meaningful connection to the flow of experiences up to that point. The experiencer needs to provide meaning to the experience retroactively.

Retention assumable remains unaffected, as patients with schizophrenia remain able to experience that something has happened, and that time is in fact moving on (Stanghellini et al., 2015; Vogel et al., 2019a). The intact retention causes events to be judged after the fact (Fuchs and Van Duppen, 2017). The experiences made do not integrate automatically, but the experiencer needs to explain them actively within their individual narrative (Vogel et al., 2019a). On the imminent scale, this accounts for the formation of delusions (Fuchs, 2007, 2013; Vogel et al., 2019a). On a larger scale, when attempting to integrate e.g., an entire psychotic episode into a biographic account, this integration accounts for coping with the illness. If it fails, it may account for depressive comorbidity (Vogel et al., 2019a) or chronified delusional systems (Fuchs, 2007, 2013).

As passive synthesis is a prerequisite to subjective intentionality (Husserl, 1928; Dainton, 2010; Fuchs, 2013), its alteration manifests itself in an externalization (Fuchs, 2007) of the intruding protentions. The experience thus affected is no longer *mine*, but must be caused by an external agent or be available to others as well. Although, this externalization has been conceptualized as a retroactive act (Fuchs, 2007), psychopathology usually determines these experiences to be “immediate” (Jaspers, 1913, p. 48; Walker, 1991), meaning that it emerges without rational or emotional reason(ing) (Broome et al., 2017). It means that no active thought is necessary to form the experience of non-mineness (Schneider, 1973, p. 124). This immediacy demonstrates that it is passive synthesis, which is affected in schizophrenia. The seemingly retro-*active* integration is in no way active, but a *passive* function of unaffected retention.

The change in experience first manifests itself in delusional moods of constant imminence (Conrad, 1958; Schneider, 1973; Fuchs, 2007; Vogel et al., 2019a). The immediacy of non-mineness and sudden meaning forms the externalized experiences such as hallucinations, delusional perceptions, and self-disorders. The necessity to cope with the profound alterations in experience leads to patients performing “delusional work” (Casey and Kelly, 2019, p. 49) and forming delusional ideas and systems. The attempts at giving meaning to the experiences occurs embedded in the individual biography, providing the content of the delusions and hallucinations. Even after remission, sense making is the wish of many patients (Vogel et al., 2019a).

We thus differentiate between the passive, immediate and automatic, alteration of experience itself, and the active, in a sense biographical, explanation of the altered experiences.

## Understanding and Explaining the Temporality of Schizophrenia

The neuroscientific and the psychopathological theories of predictive processing and temporality are an interesting example of two different methods converging on each other and complementing each other. This convergence stems from two commonalities between the aims and the results of both approaches. First, they succeed at describing mechanisms underlying the same set of symptoms. Second, they located these mechanisms in the same aspect of neural processes and experience: temporality.

The symptoms that both theories account for range from straightforward to highly complex. At this point, it is important to stress again the difference in method between the two approaches. While the scientific method aims at *explaining* the *causal* relationship between symptom and (patho-) physiology, the psychopathological method aims at *understanding* the nature of the *experience* of these symptoms (Jaspers, 1913, pp. 13 ff., pp.145 ff., and pp. 189 ff.). The scientific method proposes a chain of events triggered by a pathological process (Jaspers, 1913, pp. 189 ff.). Psychopathology describes how an experience may develop from another (Jaspers, 1913, pp.145 ff.). While it is and will become obvious that in practice these approaches necessarily overlap and contribute to each other to a certain degree, the fundamental difference in the approaches needs to be clear.

The aberrant salience hypothesis in combination with predictive processing theory proposes a causal chain starting with dopaminergic dysregulation. The dysregulation causes a false weighing of the prediction error. The enhanced prediction error then moves up the predictive hierarchy. Depending on the error's value different symptoms develop. The causal chain may be extended backward through e.g., neuroanatomical and genetic research (Lau et al., 2013; Barkus et al., 2014; Kätzel et al., 2020) or forward through e.g., explaining the effect of antipsychotics (Kapur, 2003; Lau et al., 2013; Kätzel et al., 2020). Strictly speaking, the method can only account for signs and symptoms that develop within this causal link. For example, with the current knowledge it is not possible to explain how delusional content develops, e.g., why one person feels persecuted and the other surveilled and the next both.

The psychopathology of time experience embeds psychotic symptoms within a phenomenological basis. Experience follows a temporal structure. Its alteration leads to the emergence of symptoms. The specific symptoms present themselves in accordance with the remaining experiential structure and its individual biographic enrichment. The process is not strictly causal however, as the theory does not attempt at explaining them by formulating generalizable and predictable rules as to what symptom necessarily needs to follow which change. The goal is to understand the basic change in experience by bringing to mind its implications for experience. The method cannot go beyond what the patient directly experiences and reports.

The advantage of combining both approaches becomes apparent in their limitations. Where explaining allows going beyond a patient's experience, understanding provides the individualized and empathic approach. The case presented here is particularly interesting, as they share striking commonalities. Common to both approaches is their reference to and reliance on temporality.

A break in temporal flow is responsible for the emergence of symptoms in both approaches. For psychopathology, the protentional function—the expectation of the future—breaks up and causes gaps, into which experiences intrude. The question unanswered is how these expectations change and what brings on that change. Psychopathology can only claim that expectations are present and necessary for experience as it presents itself. But how does an experiential content “intrude” into experience? How can a “gap” in temporal flow occur?

Phenomenological psychopathology alone is not readily able to answer these questions. It is by default focused on and restricted to conscious experience. Any process inaccessible by conscious experience cannot be understood (Jaspers, 1913). If we wish to know what causes protention to fail, we will have to try to explain it. We will have to turn to neuroscientific explanations.

Predictive processing theory fills the gap. We may explain failing protention in terms of the aberrant prediction error. Protention and prediction both relate to the future directedness of experience and perception. Assumably, both fail during psychosis in schizophrenia. The “gap” in temporality then corresponds to the assumption of a faulty prediction. What a appears like a missing protention—i.e. the temporal gap—can be explained as the cognitive systems “mistrust” in the imminent percept. Where we should encounter a definite prediction, we find an indefinite prediction. In other words, the “gap” corresponds to a lack of certainty.

The seemingly external experience caused by the protentional gap stems from a percept erroneously marked by dopaminergic activity. The lack of certainty encoded by the dopaminergic pulse provides the perceptual content with novelty and surprise. The resulting aberrantly salient percept penetrates the temporal field. The aberrant release of dopamine fractures the temporal flow and the intentional arch. As formulated by psychopathologists, it is the unlikely that intrudes into the fragmented temporality. The aberrant salience hypothesis complements this account by assuming that it does not only intrude but causes the fragmentation (Vogel et al., 2020b).

The fragmentation in both cases develops from the interruption of an ongoing, extended, future-directed, self-updating procedure causing a novel experience. Both accounts deal with the fragmentation of this temporal procedure similarly. They propose an explanation of the novel experiences through prior (biographical) knowledge. In effect, the remaining not primarily affected aspects of the temporal system start compensating for the smaller level disturbance and thereby account for the development of larger scale symptoms.

It is important to remember, that the two theories are not identical. Inherently, the two methods attempt at describing to different phenomena. Phenomenological and descriptive psychopathology deals with conscious experience. Clinical neuroscience wishes to uncover (patho-) physiological processes. Their combination is no biological explanation of experiences during mental illness. We believe that their combination can only be fruitful to complement the other method where it reaches its limits.

Pointing out the advantages of combining theories from different methods is further important to outline their methodological limitations. Both aberrant salience theory and fragmented time theory try to explain signs and symptoms in schizophrenia in their totality. Particularly early theories on the psychopathology of time have been criticized as idiosyncratic, too broad, and even dogmatic (Kupke, 2009; Juckel et al., 2022). Considering the heterogenous presentation and multifactorial origins of schizophrenia (for recent review see Radua et al., 2018; McCutcheon et al., 2020) this critique is certainly justified and could be extended to aberrant salience theory. Accordingly, it is important to point out, that theories from either method have their inherent limits.

Informing an understanding method with explanatory models and vice versa exemplifies the advantages and possibly necessity of methodological pluralism in psychiatric clinical practice and research (Ghaemi, 2006, 2007). For example, Jaspers (Jaspers, 1913, pp. 90 ff., pp. 148 ff.; also see Schneider, 1973, pp. 8 ff.) speaks of the limit of the phenomenological method in terms of the “incomprehensibility” of psychosis. At a certain point during the psychopathological exploration of a patient's symptoms, any further empathizing will not lead to a better understanding. What a person is able to access experientially limits what is accessible to the examiner. For example, we may understand what the experience of one's thoughts being controlled by external forces means to a person; how it makes them feel; who the controlling forces may be; what they make them do, etc. However, we may never understand where or why the experience originated; we may not even understand what the experience actually feels like (Jaspers, 1913, pp. 90 ff.). By use of explanatory models, such as e.g., predictive processing, we cannot close the fringe of incomprehensibility. However, we may be able to cross it.

## Conclusion

We presented two theories, one from psychopathology, and one from clinical neuroscience, attempting to understand and explain the symptoms of schizophrenia. Both altered time experience theory and the aberrant salience hypothesis describe changes in temporality. Psychopathology describes a fragmentation of the passive synthesis of time consciousness due to weakened protention. Clinical neuroscience proposes an interruption of predictive processes in the brain by chaotic dopamine activity. The two approaches complement each other by revealing and bridging their limitations. Aberrant salience offers potential causal processes of symptoms. We argue that percepts enriched with aberrant salience intrude into the temporal field causing the fragmentation of temporality proposed by psychopathology. Altered time experience manages to formulate an individualized account of symptomatology and helps connecting (patho-) physiological processes to experience. The temporality of aberrant salience and psychosis demonstrated that combining knowledge gained from both approaches enables researchers and clinicians to improve their understanding of patients and their experience. It offers insight that may inspire further research. It may foster future somatic and phenomenological differentiation by revealing the limitations and imprecision of isolated approaches. In the end, mixing methods may improve and inspire research theories and clinical diagnosis.

## Author Contributions

The author confirms being the sole contributor of this work and has approved it for publication.

## Conflict of Interest

The author declares that the research was conducted in the absence of any commercial or financial relationships that could be construed as a potential conflict of interest.

## Publisher's Note

All claims expressed in this article are solely those of the authors and do not necessarily represent those of their affiliated organizations, or those of the publisher, the editors and the reviewers. Any product that may be evaluated in this article, or claim that may be made by its manufacturer, is not guaranteed or endorsed by the publisher.
